# Yield, nutrition, and leaf gas exchange of lettuce plants in a hydroponic system in response to *Bacillus subtilis* inoculation

**DOI:** 10.3389/fpls.2023.1248044

**Published:** 2023-10-25

**Authors:** Carlos Eduardo da Silva Oliveira, Arshad Jalal, Jailson Vieira Aguilar, Liliane Santos de Camargos, Tiago Zoz, Bhim Bahadur Ghaley, Mostafa A. Abdel-Maksoud, Khaloud Mohammed Alarjani, Hamada AbdElgawad, Marcelo Carvalho Minhoto Teixeira Filho

**Affiliations:** ^1^ Department of Plant Protection, Rural Engineering and Soils, School of Engineering, São Paulo State University - UNESP-FEIS, Ilha Solteira, São Paulo, Brazil; ^2^ Department of Biology and Zootechnics, Lab of Plant Morphology and Anatomy/Lab Plant Metabolism and Physiology, School of Engineering, São Paulo State University - UNESP-FEIS, Ilha Solteira, São Paulo, Brazil; ^3^ Department of Crop Science, State University of Mato Grosso do Sul – UEMS, Mundo Novo, Mato Grosso do Sul, Brazil; ^4^ Department of Plant and Environmental Sciences, Faculty of Science, University of Copenhagen, Copenhagen, Denmark; ^5^ Botany and Microbiology Department, College of Science, King Saud University, Riyadh, Saudi Arabia; ^6^ Laboratory for Molecular Plant Physiology and Biotechnology, Department of Biology, University of Antwerp, Antwerp, Belgium

**Keywords:** biological nitrogen fixation, growth-promoting bacteria, *Lactuca sativa* L., photosynthetic efficiency, water use efficiency

## Abstract

Inoculation with *Bacillus subtilis* is a promising approach to increase plant yield and nutrient acquisition. In this context, this study aimed to estimate the *B. subtilis* concentration that increases yield, gas exchange, and nutrition of lettuce plants in a hydroponic system. The research was carried out in a greenhouse in Ilha Solteira, Brazil. A randomized block design with five replications was adopted. The treatments consisted of *B. subtilis* concentrations in nutrient solution [0 mL “non-inoculated”, 7.8 × 10^3^, 15.6 × 10^3^, 31.2 × 10^3^, and 62.4 × 10^3^ colony forming units (CFU) mL^−1^ of nutrient solution]. There was an increase of 20% and 19% in number of leaves and 22% and 25% in shoot fresh mass with *B. subtilis* concentrations of 15.6 × 10^3^ and 31.2 × 10^3^ CFU mL^−1^ as compared to the non-inoculated plants, respectively. Also, *B. subtilis* concentration at 31.2 × 10^3^ CFU mL^−1^ increased net photosynthesis rate by 95%, intercellular CO_2_ concentration by 30%, and water use efficiency by 67% as compared to the non-inoculated treatments. The concentration of 7.8 × 10^3^ CFU mL^−1^ improved shoot accumulation of Ca, Mg, and S by 109%, 74%, and 69%, when compared with non-inoculated plants, respectively. Inoculation with *B. subtilis* at 15.6 × 10^3^ CFU mL^−1^ provided the highest fresh leaves yield while inoculation at 15.6 × 10^3^ and 31.2 × 10^3^ CFU mL^−1^ increased shoot fresh mass and number of leaves. Concentrations of 7.8 × 10^3^ and 15.6 × 10^3^ increased shoot K accumulation. The concentrations of 7.8 × 10^3^, 15.6 × 10^3^, and 31.2 × 10^3^ CFU mL^−1^ increased shoot N accumulation in hydroponic lettuce plants.

## 1 Introduction

Lettuce, a low-calorie, low-fat, and low-sodium salad vegetable, is an abundant source of natural health-promoting phytochemicals. It is a substance-rich source of vitamins, including glycosylated flavonoids, hydroxycinnamic acids, sesquiterpene lactones (e.g., lactucin), carotenoids, B-group vitamins, vitamin C, tocopherol, and ascorbic acid. It is also rich in iron (Fe). Secondary metabolites of lettuce are potentially associated with many beneficial health properties, including anti-free radicals, anti-cardiovascular, anti-inflammatory, anti-cystic, and anti-diabetic effects (Kim et al., 2016; Yang et al., 2022).

Lettuce production is increasing worldwide, and the possibility of its production throughout the year has been a problem, especially when cultivated in the soil. In addition, root–leaf diseases during prolonged rain conditions are another factor affecting lettuce production (Opatovsky et al., 2019). The nutrient solution recirculation system in the Nutrient Film Technique (NFT) in hydroponics provides more efficient disease management in lettuce cultivation, increases plant precocity and yield, and reduces water consumption compared to the production of leafy vegetables in soil (Dalastra et al., 2020). Greater efficiency in water use is essential for the global water crisis (Kappel et al., 2021), in addition to increasing the absorption and concentration of nutrients in lettuce plants (Aini et al., 2019).

Plant growth-promoting bacteria that improve growth, nutrient acquisition, water use efficiency, and yield gains have been extensively studied in production systems under field conditions (Fukami et al., 2018). Inoculation with *Bacillus subtilis* has previously been associated with several mechanisms that promote plant growth, such as producing phytohormones and siderophores and increasing nutrient acquisition (Kang et al., 2019). In a study with the inoculation of *Bacillus* spp. in an aquaponic system with lettuce, it was possible to verify an increase in shoot and root growth, acquisition of N and P, and even improved plant quality for human consumption (Kasozi et al., 2021).

The use of *B. subtilis* in the nutrient solution (inoculation) to improve the cultivation of lettuce in hydroponics with the intent to produce healthy and quality foods, mitigate the consumption of fertilizers, and increase the yield of lettuce in hydroponic cultivation can be a valuable alternative. Owing to the scarcity of studies related to inoculation in hydroponic systems, a study to find the ideal concentration of inoculant in the nutrient solution is indispensable. In this context, this study aimed to estimate the *B. subtilis* concentration that promotes increases in yield, gas exchange, and nutrition of lettuce plants in a hydroponic system.

## 2 Materials and methods

### 2.1 Environmental characterization

**Figure 1:**
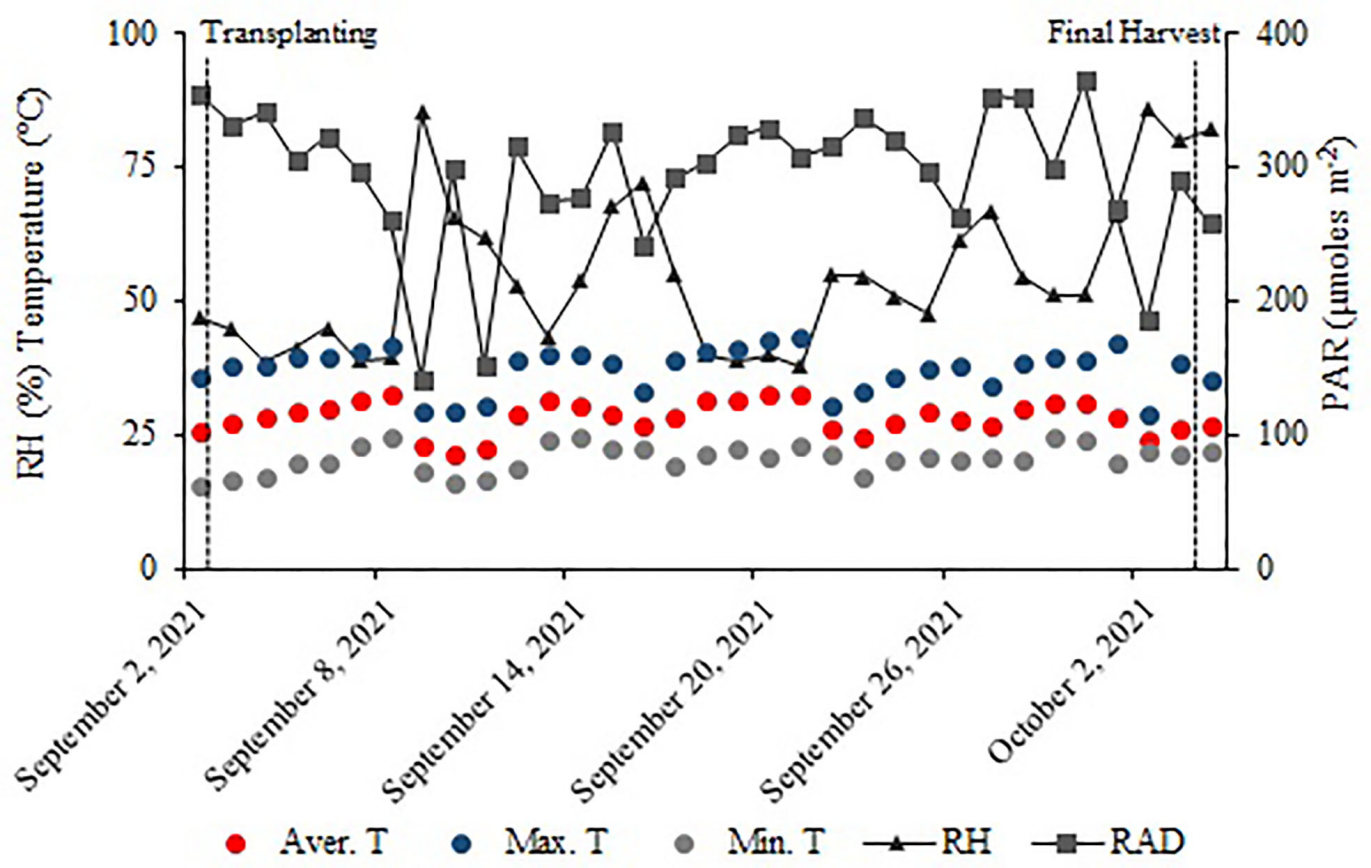
Relative air humidity (RH); maximum (Max. T), average (Avg. T), and minimum (Min. T) temperatures; and PAR radiation (RAD) during the experiment conduction.

The experiment was conducted in an NFT hydroponic lettuce cultivation system in a greenhouse with 30% shading at São Paulo State University - (UNESP), Ilha Solteira - SP, Brazil (20°25′07″ S, 51°20′31″ W, and an altitude of 376 m). Weather data were collected outside the greenhouse, in the UNESP weather station, between 2 September and 4 October 2021 (**Figure 1**).

### 2.2 Experimental design

The randomized block design with five replications was adopted. Each experimental unit consisted of four lettuce plants randomly collected on the bench in the four central channels in the hydroponic. The treatments comprised five concentrations of *B. subtilis*, strain CCTB04, in the nutrient solution, which were obtained by diluting a liquid inoculant containing 1 × 10^8^ colony forming units (CFU) mL^−1^ in the nutrient solution of the reservoirs of the hydroponic system. The following doses of inoculant were diluted in 100 L of nutrient solution: 0.0 (non-inoculated), 7.8, 15.6, 31.2, and 62.4 mL, obtaining the following concentrations of *B. subtilis*: 0.0, 7.8 × 10^3^, 15.6 × 10^3^, 31.2 × 10^3^, and 62.4 × 10^3^ CFU mL^−1^ of nutrient solution. The inoculant doses were applied in the nutrient solution only on the day of transplanting the lettuce seedlings. The isolates of *Bacillus subtilis*, strain CCTB04 were obtained from the collection of the Brazilian Agricultural Research Corporation (EMBRAPA). This isolate was sent to the UNESP microbiology laboratory for multiplication. The multiplication of bacteria was carried out in Starch Casein Agar medium (10 g of starch, 0.3 g of casein, 2 g of KNO_3_, 2 g of NaCl, 2 g of K_2_HPO, 0.05 g of MgSO_4_.7H_2_O, 0.01 g of FeSO_4_.7H_2_O, 16 g of Agar, and 1,000 mL of distilled water) and incubated at 25°C for 7 days under constant light (Bettiol et al., 2005). The colony count of the liquid inoculant was carried out using a digital colony counter (CP 600 Plus, Phoenix Ind. Com. Equipamentos Científicos Ltda, Araraquara, SP, Brazil). The colony-forming units per milliliter of the *B. subtilis*, liquid inoculant, was calculated at 1 × 10^8^ CFU mL^−1^.

### 2.3 Experimental characterization

The experiment was set up in a Nutrient Film Technique (NFT) system in individual benches of 6 m in length and 10% slope. The cultivation channels were made of PVC with a rectangular section 8 cm wide and 4 cm high and upper perforations to accommodate plants every 25 cm (**Supplementary Figure 2**). Each bench consisted of six cultivation channels 20 cm apart with an individual pumping system and a 300-L reservoir with a flow rate of 1 L per minute and continuous flow of the nutrient solution.

Iceberg lettuce, Angelina cultivar, was used. This cultivar shows vigorous growth with closed, compact, and uniform heads under cultivation in soil and hydroponic systems, moderate levels of resistance to bacteriosis, intense and bright green leaves, and safety of planting in periods of climatic oscillations (high adaptation to tropical growing conditions).

The seedlings were developed in phenolic foam for 15 days and later transplanted to the benches of the NTF system, where they remained for 31 days until harvest. The nutrient solution composed of concentrated fertilizers from Hidrogood Fert was used at a concentration of (0.666 g L^−1^) indicated for all stages of crop development, with the following nutrient concentrations: 10% N, 9% phosphorus (P), 28% potassium (K), 4.3% sulfur (S), 3.3% magnesium (Mg), 0.06% boron (B), 0.01% copper (Cu), 0.05% manganese (Mn), 0.07% molybdenum (Mo), and 0.02% zinc (Zn). Also, calcium nitrate (15.5% N and 26.5% calcium) at 0.495 g L^−1^ and Hidrogood Fert Iron EDDHA (6% iron) at 0.020 g L^−1^ were used.

Electrical conductivity (EC) and pH were measured and adjusted daily in the morning. On this occasion, the EC was readjusted to the EC determined for each cultivation bench with the replacement of fertilizers if necessary. This EC readjustment was conducted on the 1st, 11th, and 21st day after transplantation (DAT). The initial EC was 1.3 dS m^−1^; these values were increased according to the stage of cultivation and its response to fertilization. At the 11th DAT, the EC was increased to 1.5 dS m^−1^, and at the 21st DAT, it was raised to 1.7 dS m^−1^ and remained until harvest. Weekly supplementation with 20 mg L^−1^ of K_2_O (KCl) was carried out in all cultivation benches to improve the water status of the plants due to the effect of the high temperatures in the region (a fact observed in previous studies since non-supplementation caused wilting in the plants in the period of higher daily temperature). To maintain the pH between 6.0 and 6.5, sulfuric acid (25%) was used when pH was above 6.5, and sodium hydroxide (25%) when pH was below 6.0.

The EC and pH adjustment were described according to fertilizer uses (**Supplementary Figures 3A–E**). The effect of inoculation in the nutrient solution caused an increase in EC without adding nutrients and thus avoided the replacement of nutrients for a longer period. A hypothesis for the EC increase without adding nutrients is the occurrence of biological nitrogen fixation (BNF) due to the presence of *Bacillus subtilis* in the nutrient solution. The daily elevations of EC by inoculation were described (**Supplementary Figure 3F**).

### 2.4 Biometric and yield assessments

The evaluations were carried out 31 DAT the lettuce seedlings (harvest). Eight plants were collected and separated to quantify the number of leaves of each plant and the fresh matter of the root system and the shoot (in grams) using an analytical scale (0.001 kg). Then, the plants were dried in an air-forced circulation oven at 60°C for 72 h to obtain the dry matter of the root system and shoot (in grams) using a precision scale (0.001 g). Lettuce yield was estimated according to Equation 1.


(1) $$\text{LY}=\text{SFM}{\;}^{*}\text{PP}$$

LY—Lettuce yield (kg m^−2^);

SFM—Shoot fresh matter (kg plant^−1^);

PP—Plant population equal to 19.5 plants m^−2^.

### 2.5 Nutritional assessments

After drying, weighing, and grinding the plants in a Wiley-type mill, the concentrations of N, P, K, S, Ca, and Mg in the shoots and roots of lettuce at harvest (31 DAT) were determined according to the methodology of Malavolta et al. (1997). The accumulation of nutrients in the shoots and roots of the plants was calculated based on the respective dry matter and nutrient concentrations obtained. The accumulation of nutrients was estimated according to Equation 2.


(2) $$\text{NA}=\text{DM}{\;}^{*}\text{NC}$$

NA—Nutrient accumulation (g m^−2^);

DM—Dry matter (shoot or root) (kg m^−2^);

NC—Nutrient concentration of N, P, K, Ca, Mg, and S (g kg^−1^).

### 2.6 Physiological assessments

The evaluation of gas exchange was carried out in eight plants per plot at the time of harvest, between 9 a.m. and 11 a.m., in fully expanded leaves located in the middle part of the plant, using an infrared gas analyzer (Infra-Red Gas Analyzer—IRGA, model CRS300). The intercellular CO_2_ concentration (*Ci*—μmol CO_2_ mol^−1^ air^−1^), stomatal conductance (*gs*—mmol of H_2_O m^−2^ s^−1^), transpiration (*E*—mmol of H_2_O m^−2^ s^−1^), net photosynthesis rate (*A*—μmol CO_2_ m^−2^ s^−1^), and water use efficiency (*WUE*—mmol CO_2_ mol^−1^ H_2_O^−1^) were evaluated.

The third fully expanded leaf from the apex was collected between 9 a.m. and 10 a.m. to avoid large interferences of light intensity on the activity of the enzyme and leaf chlorophyll. Fresh leaf tissue samples were collected, stored in plastic bags, transported to the laboratory with ice, and washed with deionized water.

The total chlorophyll content (ChlT) was determined using the DMSO extracting agent. Leaf tissue (50 mg) was cut into 1-mm fragments and incubated in 7 mL of DMSO in the dark in a water bath at 65°C for 30 min, according to Hiscox and Israelstam (1979). After readings in the spectrophotometer, the contents of the photosynthetic pigments were calculated and expressed in mg g^−1^ fresh weight (FW). ChlT = (20.20 × ABS663) + (8.02 × ABS663).

### 2.7 Data analysis

The data of all variables presented normal distribution and homogeneous variances (Shapiro–Wilk test). The data were submitted for analysis of variance, and the significance of the mean squares obtained was tested by the *F*-test at a 5% probability level. The means relative to concentrations of *B. subtilis* were compared by the Tukey test at 5% probability.

## 3 Results

### 3.1 Yield components

**Figure 2:**
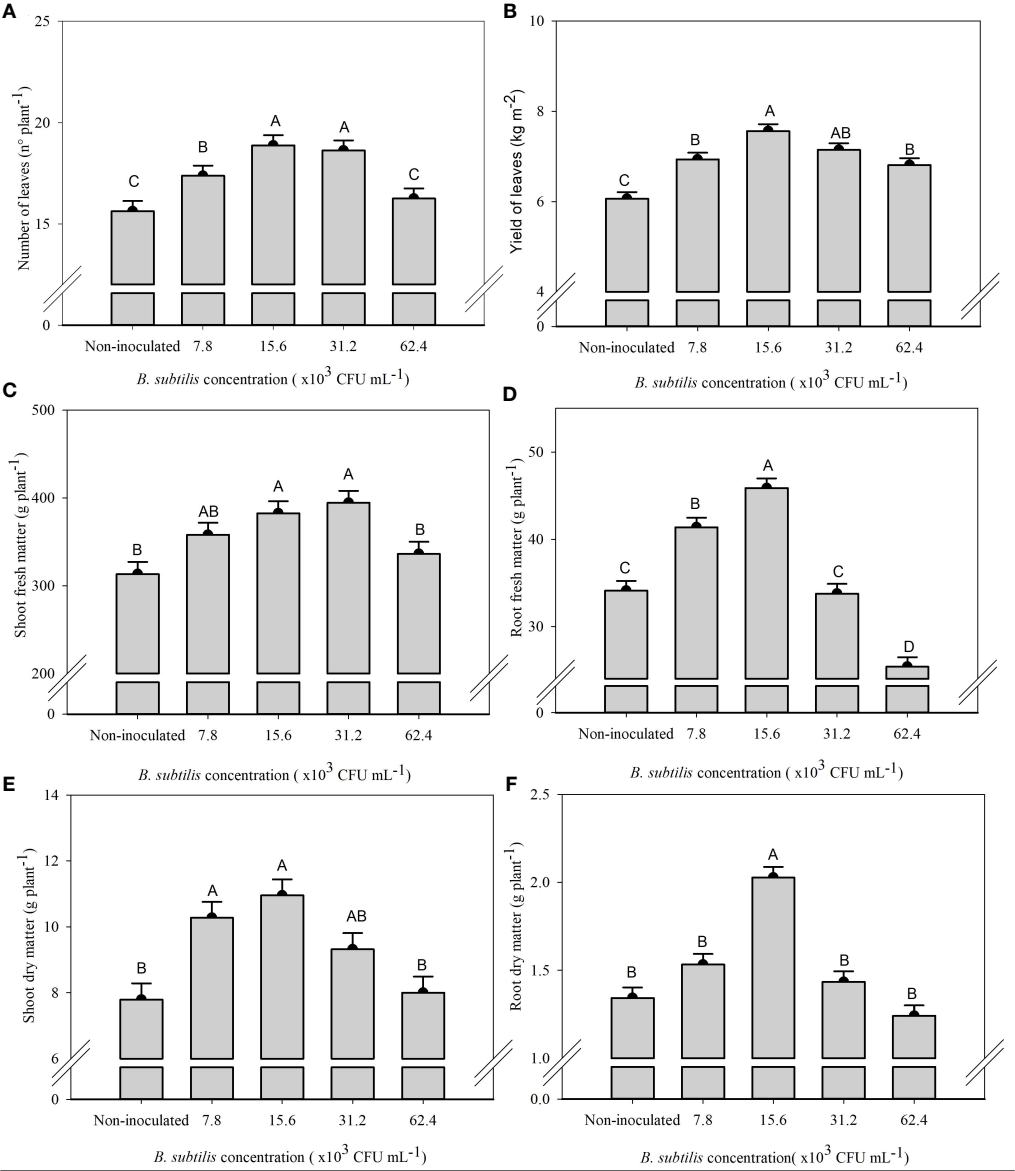
Number of leaves **(A)**, yield of fresh leaves **(B)**, fresh mass of shoot **(C)** and roots **(D)**, and dry mass of shoot **(E)** and roots **(F)** of lettuce plants in the hydroponic NFT system according to the concentrations of *Bacillus subtilis* in the nutrient solution. Different capital letters in bars are statistically different by Tukey’s test at probability ofp ≤ 0.05. Error bars indicate the standard deviation of the mean (n = 5).

There was a significant effect of *B. subtilis* on the fresh and dry matter of shoot and roots, the number of leaves, and the leaf yield of lettuce in the hydroponic system (**Supplementary Table 1**). The highest number of leaves were 18.8 and 18.6 leaves plant^−1^ at *B. subtilis* concentrations of 15.6 × 10^3^ and 31.2 × 10^3^ CFU mL^−1^ and the highest shoot fresh matter were 358, 382, and 394 g plant^−1^ at *B. subtilis* concentrations of 7.8 × 10^3^, 15.6 × 10^3^, and 31.2 × 10^3^ CFU mL^−1^, respectively. The higher number of leaves results in greater use of the plant to prepare salads (**Figures 2A, C**). The highest leaf yield (7.56 and 7.14 kg m^−2^ of fresh leaves of lettuce) occurred with the inoculation with *B. subtilis* at 15.6 × 10^3^ and 31.2 × 10^3^ CFU mL^−1^, respectively (**Figure 2B**). There was an increase of 25% and 18% in leaf yield with *B. subtilis* at 15.6 × 10^3^ and 31.2 × 10^3^ CFU mL^−1^ compared to non-inoculated plants, respectively. There was also an increase of 20% and 19% in the number of leaves at 15.6 × 10^3^ and 31.2 × 10^3^ CFU mL^−1^ compared to non-inoculated plants, respectively. A 15%, 22%, and 25% increase in shoot fresh matter with inoculation at 7.8 × 10^3^, 15.6 × 10^3^, and 31.2 × 10^3^ CFU mL^−1^ compared to non-inoculated treatment was found. Both traits are closely related to improving yield efficiency (**Figures 2A–C**).

The highest root fresh and dry matter (45.87 and 2,02 g plant^−1^) occurred with the inoculation with *B. subtilis* at 15.6 × 10^3^ CFU mL^−1^, which corresponded to an increase of 34% and 51% compared to non-inoculated treatment, respectively (**Figures 2D, F**). The highest shoot dry matter occurred at 7.8 × 10^3^, 15.6 × 10^3^, and 31.2 × 10^3^ CFU mL^−1^ with an accumulation of 10.27, 10.95, and 9.32 g plant^−1^, approximately 31%, 40%, and 20% higher than non-inoculated plants, respectively (**Figure 2E**).

### 3.2 Plant physiology

There was a significant effect of *B. subtilis* on the intercellular CO_2_ concentration (*Ci*), stomatal conductance (*gs*), net photosynthesis rate (*A*), transpiration (*E*), water use efficiency (*WUE*), and total chlorophyll content (ChlT) (**Supplementary Table 2**).

**Figure 3:**
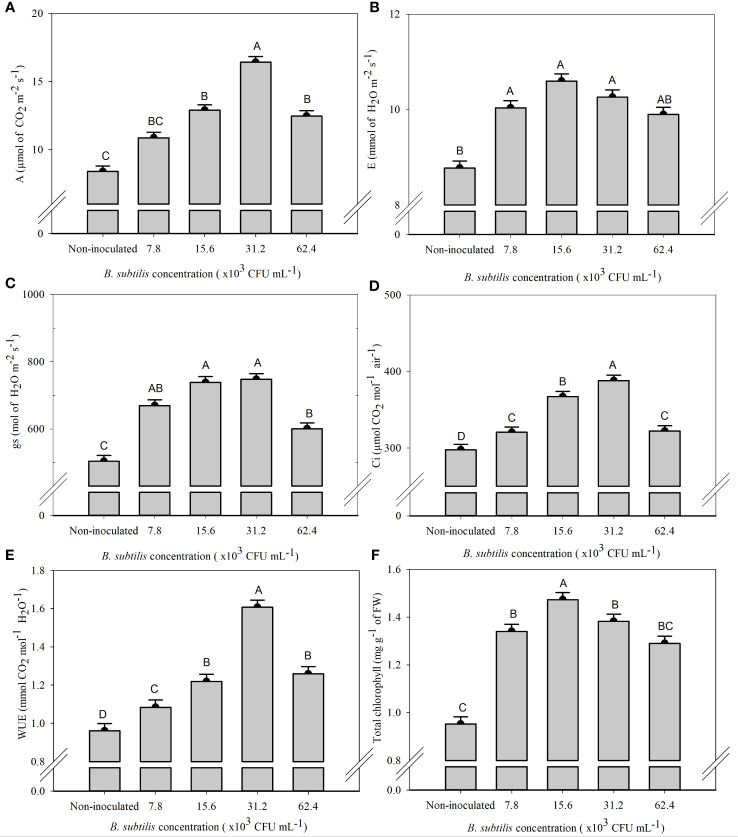
Net photosynthesis rate—*A* **(A)**, transpiration—*E* **(B)**, stomatal conductance—*gs* **(C)**, intercellular CO_2_ concentration—*Ci* **(D)**, water use efficiency—*WUE* **(E)**, and total chlorophyll content—ChlT **(F)** of lettuce plants in the hydroponic NFT system according to the concentrations of *Bacillus subtilis* in the nutrient solution. Different capital letters in bars are statistically different by Tukey’s test at probability ofp ≤ 0.05. Error bars indicate the standard deviation of the mean (n = 5).

The highest means of *A, Ci*, and *WUE* were 16.42 µmol CO_2_ m^−2^ s^−1^, 388.25 µmol CO_2_ mol^−1^ air^−1^, and 1.61 mmol CO_2_ mol^−1^ H_2_O^−1^ at a *B. subtilis* concentration of 31.2 × 10^3^ CFU mL^−1^, with an increase of 95% in *A*, 30% in *Ci*, and 67% in *WUE* compared to the non-inoculated treatment, respectively (**Figures 3A, D, E**). The highest means of *gs*, 669.62, 738.50, and 747.62 mmol H_2_O m^−2^ s^−1^, were observed at 7.8 × 10^3^, 15.6 × 10^3^, and 31.2 × 10^3^ CFU mL^−1^, with an increase of 33%, 46%, and 48% of *gs* compared to non-inoculated plants, respectively (**Figure 3C**). The highest *E* was 10.04, 10.60, and 10.26 mmol of H_2_O m^−2^ s^−1^, obtained at *B. subtilis* concentrations of 7.8 × 10^3^, 15.6 × 10^3^, and 31.2 × 10^3^ CFU mL^−1^, equivalent to an increase of 14%, 20%, and 17% compared to non-inoculated treatment, respectively (**Figure 3B**). The highest total chlorophyll concentration (ChlT) was 1.47 mg g^−1^ FW, obtained at 15.6 × 10^3^ CFU mL^−1^, approximately 54% higher than non-inoculated plants (**Figure 3F**).

### 3.3 Nutrient accumulation in lettuce plants

The supply of N in the treatment without inoculation was greater than the accumulation of N in all plants cultivated in this treatment, demonstrating the low use efficiency of nitrogen fertilizers. On the other hand, under the inoculation with *B. subtilis*, there was an increase in the acquisition of N by plants of 25% and 36% at *B. subtilis* concentrations of 7.8 × 10^3^ and 15.6 × 10^3^ CFU mL^−1^ compared to the non-inoculated treatment, respectively (**Supplementary Figure 1**). The increment of *B. subtilis* concentrations significantly affected the accumulation of N, P, K, Ca, Mg, and S in the shoot and roots (**Supplementary Table 3**).

**Figure 4:**
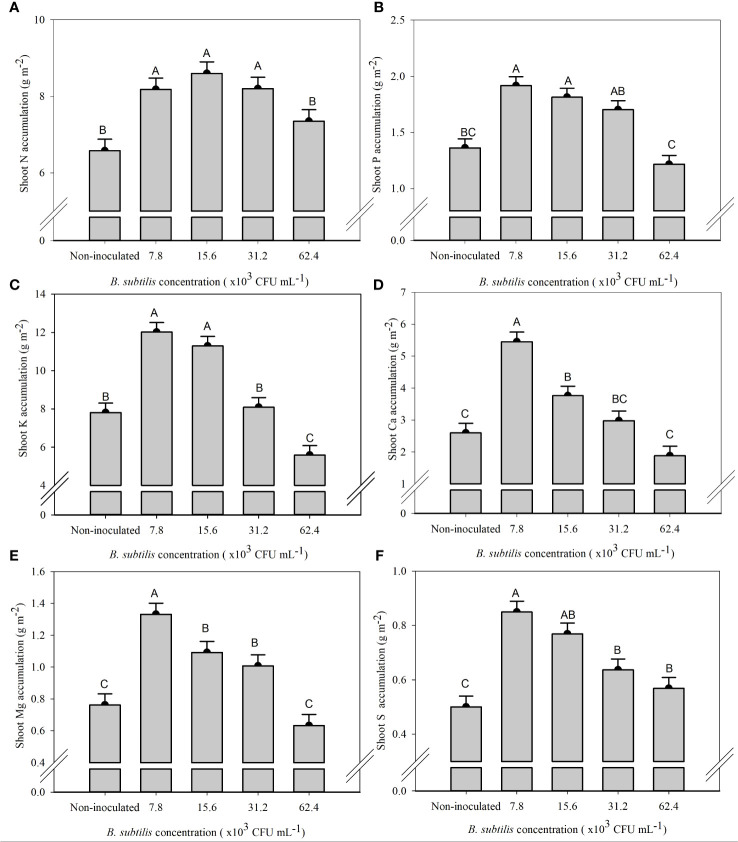
Accumulations of nitrogen **(A)**, phosphorus **(B)**, potassium **(C)**, calcium **(D)**, magnesium **(E)**, and sulfur **(F)** in the shoot of lettuce plants in the hydroponic NFT system according to the concentrations of *Bacillus subtilis* in the nutrient solution. Different capital letters in bars are statistically different by Tukey’s test at probability ofp ≤ 0.05. Error bars indicate the standard deviation of the mean (n = 5).

The highest N accumulation in the shoot was 8.18, 8.60, and 8.20 g m^−2^ at 7.8 × 10^3^, 15.6 × 10^3^, and 31.2 × 10^3^ CFU mL^−1^, with an increase of 24%, 30%, and 24% compared to non-inoculated plants, respectively (**Figure 4A**). P accumulation in the shoot was the highest at 7.8 × 10^3^, 15.6 × 10^3^, and 31.2 × 10^3^ CFU mL^−1^, with 1.91, 1.81, and 1.70 g m^−2^ of P, equivalent to an increase of 40%, 33%, and 25% compared to the non-inoculated treatment, respectively (**Figure 4B**). The concentration of 7.8 × 10^3^ CFU mL^−1^ provided the highest accumulation of Ca (5.45 g m^−2^) and Mg (1.33 g m^−2^), approximately 109% and 74% higher than non-inoculated treatment, respectively (**Figures 4D, E**). The concentrations of 7.8 × 10^3^ and 15.6 × 10^3^ CFU mL^−1^ provided the highest accumulation of K (12.01 and 11.29 g m^−2^), approximately 53% and 44% higher than the non-inoculated treatment, and S (0.85 and 0.77 g m^−2^), approximately 73% and 47% higher than the non-inoculated treatment, respectively (**Figures 4C, F**).

**Figure 5:**
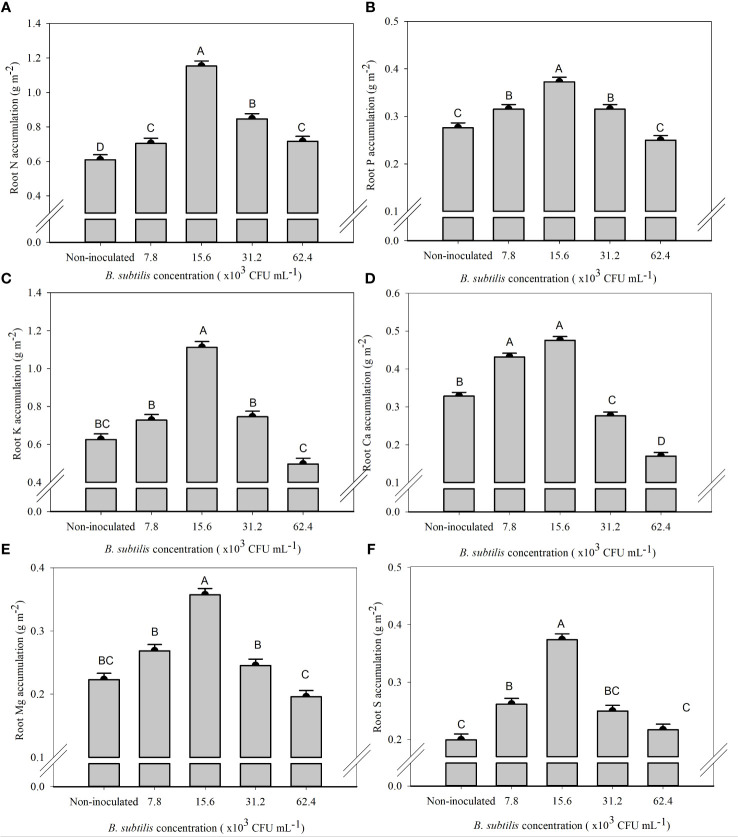
Accumulations of nitrogen **(A)**, phosphorus **(B)**, potassium **(C)**, calcium **(D)**, magnesium **(E)**, and sulfur **(F)** in the roots of lettuce plants in the hydroponic NFT system according to the concentrations of *Bacillus subtilis* in the nutrient solution. Different capital letters in bars are statistically different by Tukey’s test at probability ofp ≤ 0.05. Error bars indicate the standard deviation of the mean (n = 5).

The *B. subtilis* concentration of 15.6 × 10^3^ CFU mL^−1^ provided the highest accumulation of N, P, K, Mg, and S in the roots, with means of 1.15, 0.37, 1.11, 0.36, and 0.37 g m^−2^, and an increase of 89%, 34%, 77%, 60%, and 87% compared to non-inoculated treatment, respectively (**Figures 5A–C, E, F**). The Ca accumulation in the roots was 0.43 and 0.47 g m^−2^ at 7.8 × 10^3^ and 15.6 × 10^3^ CFU mL^−1^, with an increase of 31% and 44% in Ca accumulation of root compared to the non-inoculated (**Figure 5D**).

## 4 Discussion

The increase in root growth due to inoculation with the genus *Bacillus* in soil cultivation may be explained by several mechanisms including an increase in the biosynthesis of phytohormones and organic compounds, increased availability of nutrients, and the ability to colonize the root system of the plant (Tsotetsi et al., 2022). In our studies, in a hydroponic system, these mechanisms may explain the increase in fresh and dry matter of roots at a *B. subtilis* concentration of 15.6 × 10^3^ CFU mL^−1^ compared to the non-inoculated plants, respectively (**Figures 2D, F**). Similar results were verified in arugula plants inoculated with 10 mL 100 L^−1^ of *B. subtilis* in a hydroponic system, with a 31% increase in fresh and dry matter of arugula plants (Oliveira et al., 2023b). The greater root growth with inoculation of *B. subtilis* occurred by stimulating the increase in the production of phytohormones such as indole-3-acetic acid (IAA) by the plants, which act mainly as stimulators of root growth and, consequently, improve the exploitation of the environment of cultivation and absorption of water and nutrients (Kumari et al., 2018).

The highest shoot fresh mass, number of leaves, and fresh leaf yield of lettuce plants were noted at *B. subtilis* concentrations of 15.6 × 10^3^ and 31.2 × 10^3^ CFU mL^−1^ (**Figures 2A–C**). It has been reported that fresh mass of lettuce is one of the most important marketable traits that increase profitability (Dalastra et al., 2020; Oliveira et al., 2023c). In other studies, inoculation via nutrient solution at 10 mL 100 L^−1^ of *A. brasilense* provided an increase of 12% and 20% in the number of leaves and 23% and 32% in the leaf yield of lettuce and arugula plants, respectively (Oliveira et al., 2023a). Similar results were verified in arugula plants inoculated with 10 mL 100 L^−1^ of *B. subtilis* in a hydroponic system (Oliveira et al., 2023b). Inoculation with *B. subtilis* favors the increase in the shoot fresh and dry matter by increasing photosynthetic efficiency, carbon assimilation, and nutrient acquisition by the roots (Fonseca et al., 2022).

Leaf growth occurs with higher net assimilation of CO_2_ in the photosynthetic process that allows the formation of new leaves and an increase in plant mass, resulting in higher yield (Fonseca et al., 2022). The present results stated that inoculation with *B. subtilis* at a concentration of 31.2 × 10^3^ CFU mL^−1^ increased *A*, *Ci*, and *WUE*, while ChlT of lettuce leaves increased at a concentration of 15.6 × 10^3^ CFU mL^−1^ (**Figures 3A, D–F**). The higher water use efficiency is related to the greater integrity of leaf cell membranes, which is related to the ability of plants to keep their metabolic and photosynthetic activities in operation even in periods with high temperatures, as in the case of the present study (**Figure 1**). The greater water use efficiency provides greater turgor in the guard cells, keeping the stomata open. The *gs* was higher at *B. subtilis* concentrations of 15.6 × 10^3^ and 31.2 × 10^3^ CFU mL^−1^ compared to non-inoculated plants (**Figure 3C**). The suitable water status of plant leaves can increase stomatal opening periods, which favors photosynthetic activity and net CO_2_ assimilation by plants, and can improve the efficiency of water use and plant growth (Buckley, 2019).

An adequate supply of *B subtilis* led to increased growth of lettuce shoots and roots via enhanced nutrient uptake. At inoculation concentrations ranging from 7.6 to 31.2 × 10^3^ CFU mL^−1^, increased accumulation of N, P, Ca, Mg, K, and S (depending on the individual nutrient) was observed in the shoot and roots of lettuce as compared to non-inoculated plants (**Supplementary Table 3**; **Figures 4**, **5**).

The increase in the efficiency of nutrient uptake and transport by plants inoculated with *B. subtilis* is mainly related to increased root growth, production of siderophores, and production of secondary compounds and phytohormones in the plant (Qiao et al., 2017; Fonseca et al., 2022). Root growth also favors the increased absorption efficiency of N, P, K, Ca, Mg, and S, and the gibberellin production as previously reported in Chinese cabbage plants under inoculation with *B. subtilis* compared to non-inoculated plants (Kang et al., 2019). The increase in the accumulation of these nutrients was also observed in sugarcane under inoculation with *B. subtilis* (Fonseca et al., 2022). Other research with foliar inoculation with *T. harzianum* and *A. brasilense* in lettuce and arugula in the hydroponic system provided greater root growth and, consequently, greater acquisition of nutrients by the plants, in addition to providing less use of fertilizers (Moreira et al., 2022; Oliveira et al., 2022; Gato et al., 2023). Inoculation with *B. subtilis* via nutrient solution in arugula plants provided an increase of 6%, 43%, 36%, 52%, and 45% in the accumulation of N, K, Ca, Mg, and S in the shoots, respectively, and provided an increase of 32%, 107%, 6%, 65%, and 19% in the accumulation of N, P, K, Ca, and S in the roots, respectively (Oliveira et al., 2023b).

There was increase in ChlT content with the inoculation of *B. subtilis* at 15.6 × 10^3^ CFU mL^−1^ (**Figure 3F**). Photosynthetic CO_2_ assimilation rates are favored by adequate K and Mg nutrition, increasing intercellular CO_2_ levels and leaf photosynthetic activity (Singh and Reddy, 2017). Because of the osmotic role of K in increasing leaf stomatal conductance (acting in guard cell regulation), lower K uptake impairs photosynthesis (Jákli et al., 2017). Plant growth and metabolism require the translocation of carbohydrates from photosynthetically active tissues; sucrose loading is the main form of carbohydrate transport in plants, and K and Mg deficiency in the phloem can impair the efficient transport of carbon to within the plant, and adequate supply of these two nutrients enhances photoprotection (Tränkner et al., 2018). Ca^2+^ binding proteins are chloroplast residents, but some are located in the chloroplast membrane, such as the transporter-like S-adenosylmethionine (Stael et al., 2011). Ca^2+^ acts as a cofactor for the activation of redox enzymes related to photosystem II membranes; Ca^2+^ also regulates the enzymes fructose-1,6-bisphosphatase (FBPase) and sedoheptulose-1,7-bisphosphatase (SBPase) that are fundamental in the Calvin cycle in the carbon assimilation pathway that occurs in the chloroplast stroma (Wang et al., 2019).

The use of inoculation with *B. subtilis* has been highlighted as promoting plant growth, increasing crop yield, and increasing efficiency in nitrogen acquisition. The main mechanisms related to greater nitrogen acquisition caused by *B. subtilis* are greater root growth (increases soil exploitation by roots), growth promotion (increased production of secondary compounds and phytohormones), and biological nitrogen fixation (increases nitrogen metabolism activity and yield by increasing carbon accumulation) (Hashem et al., 2019). At *B. subtilis* concentrations of 7.8 × 10^3^ and 15.6 × 10^3^ CFU mL^−1^, there was higher N accumulation than in the non-inoculated treatment (**Supplementary Figure 1**), which may occur due to the effect of biological N fixation by *B. subtilis* since the only nitrogen source available for the roots in the hydroponic system is from the nutrient solution. Inoculation with *B. subtilis* in the nutrient solution may allow a symbiotic interaction with the plant, permitting *B. subtilis* to sequester N from the atmosphere via nitrogen fixation and provide it to the plant. In studies with several species of *Bacillus*, the presence of the *nif* and *fixam* genes, responsible for the ability to perform nitrogen fixation, were confirmed by genome sequencing. In addition, these *Bacillus* species increased biological N fixation in a tropical region (Yousuf et al., 2017). *B. subtilis* provides greater nitrogen use efficiency and mitigation of ammonia emission to the environment due to the ease of acquisition of ammonium molecules derived by bacteria during the nitrogen fixation process and its transport to plant tissues (Sun et al., 2020). In addition, according to Gunka and Commichau (2012), inoculation with *B. subtilis* increased the *nrg*A gene expression related to the uptake of N by plants in the ammoniacal form, inducing a greater amount of ammonium available through the process of biological N fixation.

Inoculation with *B. subtilis* increased plant mass accumulation, nutrient accumulation, and photosynthetic and water use efficiency. The main mechanisms of *B. subtilis* to improve the growth, nutrition, and leaf gas exchange of hydroponic lettuce plants are related to increased efficiency in nutrient uptake and photosynthesis. In addition, it was possible to verify alterations in the nutrient solution, raising the hypothesis that there is an interaction between *B. subtilis* and roots in the hydroponic system, resulting in biological nitrogen fixation. This hypothesis will require further research to confirm the presence of nitrogen fixation genes in this *B. subtilis* strain and their expression under hydroponic growth conditions with lettuce. However, increasing lettuce leaf yield with *B. subtilis* inoculation is a low-cost technology that reduces fertilizer consumption and increases farmer profitability.

## 5 Conclusions

The *B. subtilis* concentration at 15.6 × 10^3^ and 31.2 × 10^3^ CFU mL^−1^ in the nutrient solution is indicated to achieve the highest yield efficiency in the hydroponic system since it provided the highest lettuce fresh leaf yield, shoot fresh matter, and number of leaves. *B. subtilis* inoculation at 15.6 × 10^3^ CFU mL^−1^ increased root fresh and dry mass in lettuce plants grown in the hydroponic system.

Inoculation with *B. subtilis* at 7.8 × 10^3^ CFU mL^−1^ in the nutrient solution increased the accumulation of Ca, Mg, and S in the shoot, and at 7.8 × 10^3^ and 15.6 × 10^3^ CFU mL^−1^, it increased K accumulation in the shoot. *B. subtilis* at 7.8 × 10^3^, 15.6 × 10^3^, and 31.2 × 10^3^ CFU mL^−1^ increased N accumulation in the shoot in the hydroponic system. In addition, the inoculation with *B. subtilis* at 15.6 × 10^3^ CFU mL^−1^ increased the accumulation of N, P, K, Mg, and S in the roots of lettuce plants.

*B. subtilis* at 31.2 × 10^3^ CFU mL^−1^ increased water use efficiency, intercellular CO_2_ concentration, and net photosynthesis rate of lettuce plants in the hydroponic system. *B. subtilis* at 15.6 × 10^3^ and 31.2 × 10^3^ CFU mL^−1^ increased stomatal conductance. Also, the highest total chlorophyll content was found at 15.6 × 10^3^ CFU mL^−1^ in the lettuce plants grown in a hydroponic system.

## Data availability statement

The original contributions presented in the study are included in the article/**Supplementary Materials**. Further inquiries can be directed to the corresponding author.

## Author contributions

CO and MT conceived and designed the research. CEO and JA carried out the experiment. CO and MT analyzed the data. CO wrote the manuscript. BG, MA-M, KA, and HA contributed to formal analysis and data curation. AJ, LC, and MT reviewed and translated the manuscript. All authors contributed to the article and approved the submitted version.
