# Supplementary material for: Yield, nutrition, and leaf gas exchange of lettuce plants in a hydroponic system in response to Bacillus subtilis inoculation
Source: Front Plant Sci. 2023 Oct 25;14:1248044. doi: 10.3389/fpls.2023.1248044 (PMC10634435; doi:10.3389/fpls.2023.1248044)
Supplement: Supplementary file 1 [file DataSheet_1.docx]

Supplementary Material

Yield, nutrition, and leaf gas exchange of lettuce plants in a hydroponic system in response to *Bacillus subtilis* inoculation

**Carlos Eduardo da Silva Oliveira^1^, Arshad Jalal^1^, Jailson Vieira Aguilar^2^, Liliane Santos de Camargos^2^, Tiago Zoz^3^, Bhim Bahadur Ghaley^4^, Mostafa A. Abdel-Maksoud^5^, Khaloud Mohammed Alarjani^5^, Hamada AbdElgawad^6^, Marcelo Carvalho Minhoto Teixeira Filho^1*^**

*** Correspondence:** Corresponding Author: [mcm.teixeira-filho@unesp.br](mailto:mcm.teixeira-filho@unesp.br) , +55(18)37431940

Supplementary Data

# Supplementary Figures and Tables

## Supplementary Figures

**
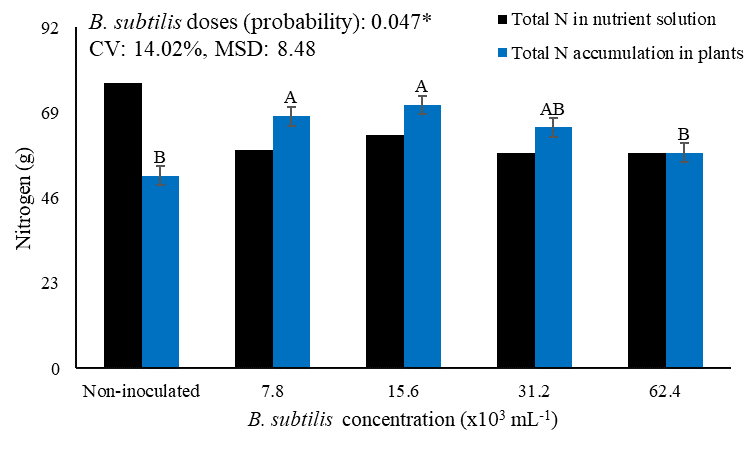
**

**Figure S1.** Total nitrogen accumulation in lettuce plants in the hydroponic cultivation bench and total nitrogen supply via nutrient solution throughout the growing period according to the concentrations of *Bacillus subtilis* via nutrient solution. Probability – P value. MSD – minimal significant difference.


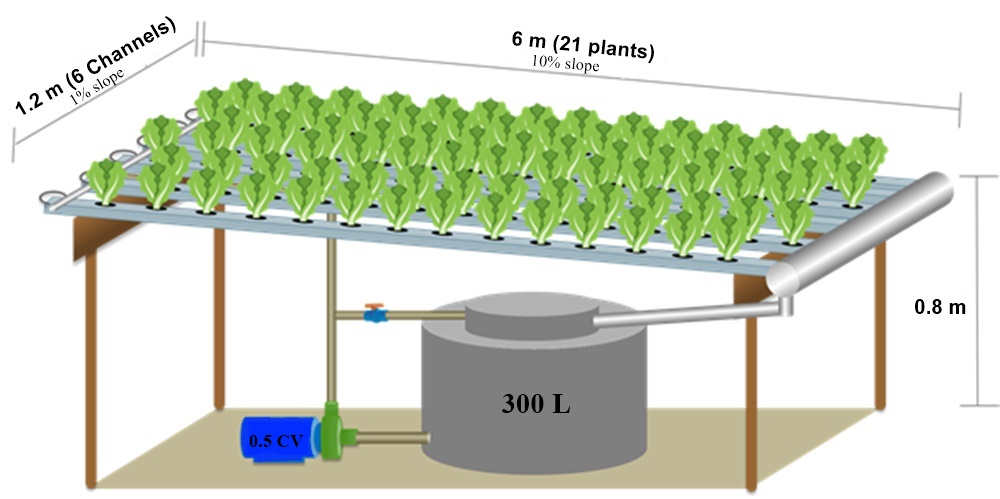


**Figure S2.** Schematic of the hydroponics bench used in the test conduction with lettuce plants. Ilha Solteira - SP, Brazil, 2021.

**
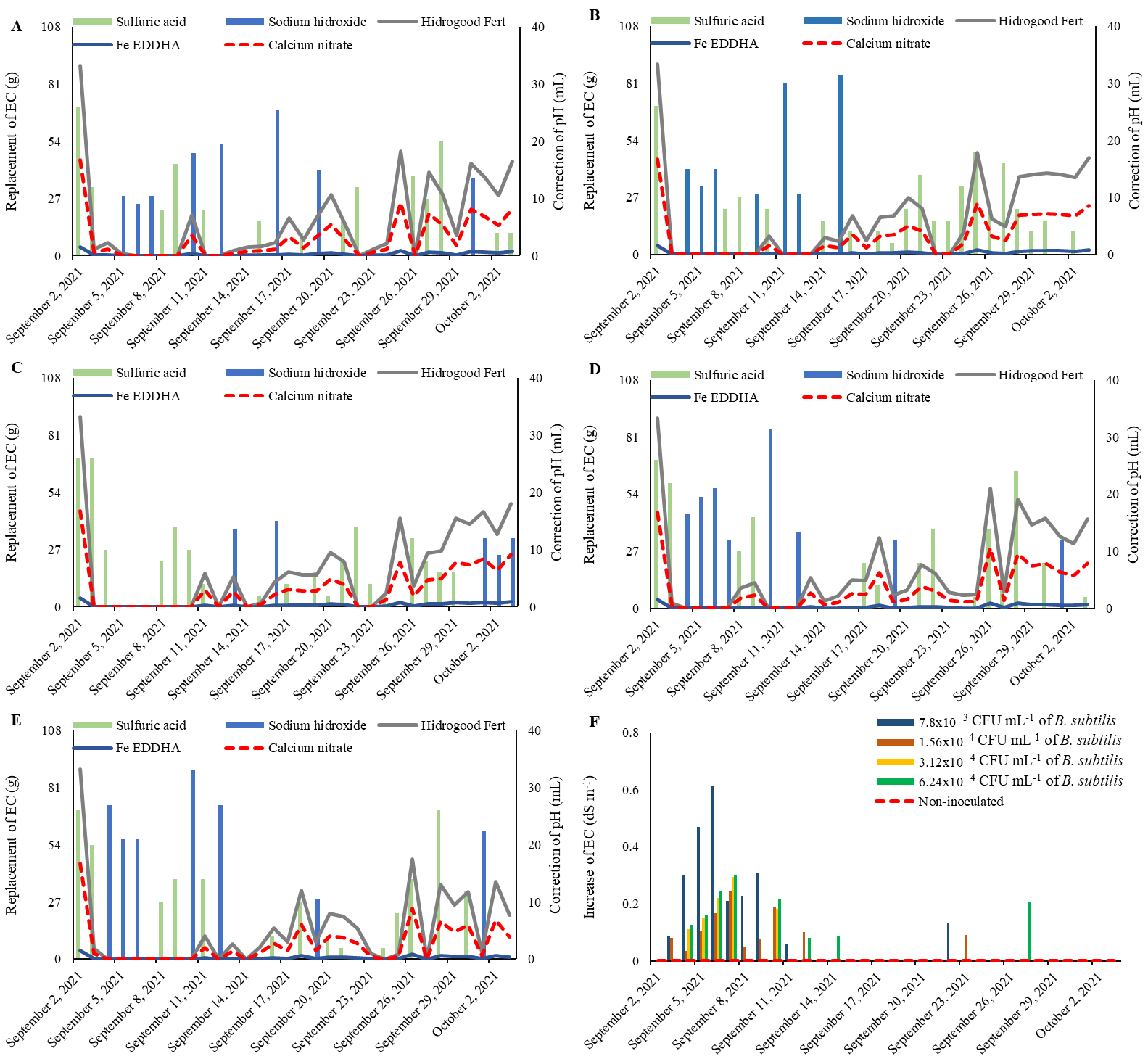
**

**Figure S3.** Replacement of pH and electrical conductivity (EC) in NFT hydroponic system in the control treatment (A), with the inoculation of *Bacillus subtilis* via nutrient solution at concentrations of 7.8x10^3^ CFU mL^-1^, (B) 15.6x10^4^ CFU mL^-1^ (C), 31.2x10^4^ CFU mL^-1^ (D), 62.4x10^4^ CFU mL^-1^ (E), and the increase in EC caused by inoculation according to each treatment (F) in the experiment period.

## Supplementary Tables

**Table S1.** Summary of analysis of variance of shoot fresh matter (SFM), root fresh matter (RFM), shoot dry matter (SDM), root dry matter (RDM), number of leaves (NL), and yield (YLD) of lettuce plants.

| Source of variation | Mean Squares | | | | | |
| --- | --- | --- | --- | --- | --- | --- |
|  | SFM | RFM | SDM | RDM | NL | YLD |
| Block | 220.78 | 11.21 | 1.52 | 0.05 | 0.75 | 0.03 |
| Concentration | 4383.1** | 201.55** | 7.63** | 0.37** | 8.14* | 2.03** |
| Residue | 757.4 | 4.89 | 0.98 | 0.02 | 1.15 | 0.09 |
| CV (%) | 7.71 | 9.61 | 10.66 | 9.21 | 6.17 | 8.76 |
| MSD | 55.05 | 1.41 | 2.21 | 0.41 | 1.12 | 0.50 |

** significant at 1%, * significant at 5%, ^ns^ not significant, CV - coefficient of variation, MSD – minimal significant difference.

**Table S2.** Summary of analysis of variance of intercellular CO_2_ concentration (*Ci*), stomatal conductance (*gs*), net photosynthesis rate (*A*), transpiration (*E*), water use efficiency (*WUE*), and total chlorophyll content (ChlT) of lettuce plants.

| Source of variation | Mean Squares | | | | | |
| --- | --- | --- | --- | --- | --- | --- |
|  | *Ci* | *gs* | *A* | *E* | *WUE* | ChlT |
| Block | 94.65 | 2504.6 | 0.11 | 0.30 | 0.007 | 0.003 |
| Concentration | 5561.2** | 41541.6** | 34.60** | 1.90** | 0.24** | 0.158** |
| Residue | 61.41 | 1821.9 | 0.87 | 0.16 | 0.01 | 0.003 |
| CV (%) | 7.31 | 8.55 | 10.65 | 9.32 | 9.62 | 8.22 |
| MSD | 17.10 | 90.01 | 2.09 | 1.15 | 0.14 | 0.10 |

** significant at 1%, * significant at 5%, ^ns^ not significant, CV - coefficient of variation, MSD – minimal significant difference.

**Table S3.** Summary of analysis of variance of nitrogen (N), phosphorus (P), potassium (K), calcium (Ca), magnesium (Mg), and sulfur (S) accumulation in shoots and roots of lettuce plants.

| Source of variation | Mean Squares - Shoot accumulation | | | | | |
| --- | --- | --- | --- | --- | --- | --- |
|  | N | P | K | Ca | Mg | S |
| Block | 0.22 | 0.047 | 5.86 | 1.76 | 0.038 | 0.013 |
| Concentration | 2.64** | 0.363** | 28.25** | 7.46** | 0.305** | 0.083** |
| Residue | 0.24 | 0.05 | 3.45 | 0.88 | 0.040 | 0.065 |
| CV (%) | 6.28 | 13.99 | 20.74 | 28.11 | 20.66 | 12.13 |
| MSD | 1.18 | 0.20 | 3.20 | 1.61 | 0.22 | 0.09 |
| Source of variation | Mean Squares - Root accumulation | | | | | |
|  | N | P | K | Ca | Mg | S |
| Block | 0.0007 | 0.0007 | 0.003 | 0.0003 | 0.0014 | 0.00034 |
| Concentration | 0.18** | 0.009** | 0.21** | 0.059** | 0.015** | 0.019** |
| Residue | 0.002 | 0.0004 | 0.004 | 0.0007 | 0.0004 | 0.0005 |
| CV (%) | 6.67 | 6.84 | 8.50 | 7.92 | 7.87 | 8.94 |
| MSD | 0.10 | 0.03 | 0.09 | 0.04 | 0.04 | 0.02 |

** significant at 1%, * significant at 5%, ^ns^ not significant, CV - coefficient of variation, MSD – minimal significant difference.
